# Thrombosis Prevention without Anticoagulation

**DOI:** 10.3389/fmed.2014.00012

**Published:** 2014-05-09

**Authors:** Alvin H. Schmaier

**Affiliations:** ^1^Division of Hematology and Oncology, Department of Internal Medicine, University Hospitals Case Medical Center, Case Western Reserve University, Cleveland, OH, USA

**Keywords:** thrombosis, fibrinolysis, factor XII, mas receptor, prolylcarboxy-peptidase, anticoagulation

The ultimate goal for hematologists, cardiologists, and vascular medicine physicians is to find agents that prevent thrombosis without creating defects in hemostasis (the cessation of bleeding). To a great extent, this notion was considered an impossible dream. However, recent advances in understanding of the plasma contact activation, kallikrein/kinin, renin angiotensin, and other vasoregulatory systems make these dreams seem feasible. Presently, all anticoagulants prevent thrombosis by interfering with hemostasis. Physicians in clinical medicine are presently excited with the recent approvals by national regulatory agencies of several target-specific oral anticoagulants (TSOACs). These agents make the lives of patients who need anticoagulants much easier. However, the targets of the TSOACs, factors IIa and Xa, still increase bleeding risk. In fact, some of these agents are associated with specific organ sensitivity to bleeding not seen with warfarin or heparin-derived agents. Furthermore, although in late development, antidotes to these agents are not present and bleeding such as into the brain can be catastrophic. We can do better than the current array of agents in practice and about to be approved for clinical practice for thrombosis protection. In recent years, investigators have learned that many proteins influence thrombosis risk without altering the hemostatic system. For example, proteins like heme oxygenase, C-reactive protein, glutathione peroxidase-3 deficiency, peptidylarginine deiminase 4, and myeloid-related protein-14 (S100A9) alter arterial or venous thrombosis propensity in mice without influence on hemostasis (1–5). Additionally, there are several targets in the contact activation (CAS), kallikrein/kinin (KKS), and renin angiotensin (RAS) systems that influence thrombosis without causing bleeding. Better understanding of the mechanism(s) by which these proteins influence venous and arterial thrombosis may very well allow for additional targets to be recognized for the development of safer therapies. The next frontier in drug development to prevent or treat thrombosis is to translate the mechanistic observations on proteins that alter thrombosis risk without bleeding into therapeutic agents.

Where do we start on this endeavor? It is already happening. For example, evidence indicates that the proteins of the plasma CAS and KKS [factor XII (XII), prekallikrein (PK), high molecular weight kininogen (HK)] influence thrombosis risk even though it is well recognized that their deficiencies in humans have no influence on hemostasis. Factor XII, PK, HK, and bradykinin B2 receptor-deleted mice are protected from venous and arterial thrombosis (6–9). More complete understanding of the mechanism of the thrombosis protection of the proteins of the CAS and KKS *in vivo* should provide leads for novel targets for development of antithrombotics. Contact activation is the process where XII and to a lesser extent PK or factor XI autoactivate into their enzymes on a surface, usually negatively charged but can be neutrally charged. Formed XIIa activates PK to plasma kallikrein that then reciprocally activates XII and they amplify each other’s activation leading to factor XI activation. It is the formation of factor XIa that initiates hemostasis after XII activation. The KKS is formed by plasma kallikrein cleaving HK to liberate bradykinin. This pathway initiates angioedema and inflammation, not thrombosis. XII autoactivation alone is a slow process but its rate of activation is markedly accelerated when HK and PK are present. Amplification of XII and PK activation when both are present occurs very rapidly especially with HK present. Contact activation occurs *in vivo* when blood interacts with artificial surfaces such as an extracorporeal circulation system, indwelling intravenous catheters, infectious organisms, and adult respiratory distress syndrome (10–13). All these states lead to thrombin formation. C1 inhibitor deficiency states or C1 inhibitor consumption by a mutant factor XIIa activate the KKS and lead to angioedema and inflammation, not thrombin formation (14, 15).

If contact activation is prevented, can thrombosis be thwarted? Antisense oligonucleotides to XII, PK, and factor XI prevent thrombosis in various arterial and venous thrombosis models (16, 17). Factor XI is at times considered a contact system protein because it is activated by XIIa. Reduction of factor XI by 50% reduces thrombosis risk in murine and baboon models without excess bleeding, but severe factor XI deficiency upon injury is a bleeding state (17). Additionally, a recent report by Renne and his associates suggests that thrombosis prevention by XIIa inhibition may be feasible on artificial surfaces such as that used in extracorporeal membrane oxygenation (18). These investigators produced a very specific antibody (3F7) to activated factor XII (XIIa) by phage display. This antibody only blocks XIIa (αXIIa) or βXIIa (Hageman factor fragment) interfering with PK or factor XI activation. In an extracorporeal circulation experiment with rabbits attached to an infant’s membrane oxygenator, 3F7 was nearly as good as unfractionated heparin to prevent d-dimer elevation, fibrinogen consumption, prothrombin 1 + 2 fragments, platelet consumption, and reduced pressures in the circuit (18). Furthermore, it did this without any increase in bleeding. Thus, precise factor XIIa inhibition alone is able to prevent thrombosis of blood when coursing through an artificial circuit such as cardiopulmonary bypass attached to an individual (19). Furthermore, such an agent needs no antidote because its presence does not interfere with tissue factor- and/or FXIa-induced hemostasis. Thus, anticoagulation without influence on bleeding is presently achievable in situations where human tissue and blood are exposed to artificial surfaces such as cardiopulmonary bypass, indwelling catheters, artificial hearts, and other devices interfaced with the vasculature. Can these observations be translated to other situations of pathophysiologic contact activation? An agent such as 3F7 also may reduce contact activation-induced sepsis from any cause, ARDS, and some attacks of hereditary angioedema.

Are there other instances whereby interruption of thrombotic mechanisms has potential to decrease arterial thrombosis risk without effect on hemostasis? These possibilities exist, but further development is needed. Recent studies from our laboratory have described the bradykinin B2 receptor-deleted mouse (*Bdkrb2^−/−^*) to be protected from thrombosis (9, 20). When we embarked upon these studies, we had hypothesized that *Bdkrb2^−/−^* mice would be prothrombotic because bradykinin is a potent stimulator of NO and prostacyclin production and tPA liberation. When we examined mechanism for thrombosis protection in *Bdkrb2^−/−^* mice, we observed increased plasma bradykinin, its breakdown product bradykinin-(1–5), and angiotensin II. Angiotensin-converting enzyme (kininase II) degrades both bradykinin and angiotensin I. Normally angiotensin II binds to its receptor, the angiotensin receptor 1, to induce vasoconstriction, elevation of blood pressure, and increase thrombosis propensity through expression of tissue factor and plasminogen activator inhibitor. However, we found in *Bdkrb2^−/−^* mice that there also was increased expression of the angiotensin receptor 2 (AT2R) that scooped up the excess angiotensin II to produce the dominant phenotype. Stimulation of the AT2R results in elevated NO and prostacyclin. In fact these animals have a long bleeding time due to prostacyclin elevation, not NO. If *Bdkrb2^−/−^* mice are treated with an antagonist to the AT2R, a cyclooxygenase inhibitor or an eNOS antagonist, the bleeding and thrombosis times shorten to normal. Thus, higher angiotensin II in the presence of increased expression AT2R has potential as antithrombotic agents. AT2R elevation lengthens thrombosis time in the presence of excess angiotensin II (9, 20).

Most would agree that elevating angiotensin II is not attractive as an anticoagulant mechanism. If we treat *Bdkrb2^−/−^* with losartan or telmisartan, angiotensin receptor 1 antagonists, we lower plasma angiotensin II levels without correcting their prolonged thrombosis time. These data suggest that there is an additional thromboprotective mechanism in *Bdkrb2^−/−^* mice. Further studies show that the angiotensin II breakdown product, angiotensin-(1–7) and its G-protein coupled receptor Mas are also over-expressed (20). If *Bdkrb2^−/−^* mice are treated with a Mas antagonist, A-779, their thrombosis times correct to normal. *Bdkrb2^−/−^* mice have prostacyclin levels threefold normal and it is associated with long tail bleeding times. These animals also have a GPVI activation and an integrin spreading defect. Recent preliminary studies in our laboratory suggest that PK-deficient mice (*Klkb1^−/−^*) have a similar mechanism for thrombosis protection. These combined data suggest that regulation of Mas expression and its influence on plasma prostacyclin levels may be sufficient for thrombosis protection without influence on hemostasis (21). Thus, regulating levels of expression of the receptor Mas alone may be sufficient to modulate thrombosis risk.

Are there additional targets for antithrombotic agent development? Prolylcarboxy peptidase (PRCP) is a serine protease that cleaves peptides when a proline is in the penultimate position of the carboxy terminus and activates PK. Its peptide substrates include angiotensin II, bradykinin-(1–8), and α-melanocyte stimulating hormone 1–13 (α-MSH_1–13_). A PRCP gene trap mouse (PRCP^gt/gt^) that has ~25% PRCP levels is lean, hypertensive, and has a propensity for arterial thrombosis. The major mechanistic feature of this animal with a thrombosis propensity is that there is increased reactive oxygen species (ROS) in its vessels and kidney. When treated with antioxidants, the ROS lowering corrects the arterial thrombosis time on two models to normal (22). The consequence of increased ROS in the vasculature of these animals is reduction of the vasoprotective transcription factors Kruppel-like factors 2 and 4 leading to reduced eNOS mRNA and uncoupled NO and less thrombomodulin activity, mRNA, and antigen. ROS oxidizes a critical methionine on thrombomodulin that interferes with protein C activation. There is also elevated tissue factor and PAI-1 in these vessels. These combined findings alter vascular health. PRCP-deficient cells and PRCP^gt/gt^ mice have reduced cell proliferation and migration, angiogenesis, and wound repair with increased apoptosis (23). They have excessive vascular inflammation after injury that is blocked genetically by concurrent deletion of the inflammatory protein, myeloid-related protein 8–14 (S100A9) (5, 23). PRCP’s growth factor domain that produces its cell proliferation and migration and angiogenesis to reduce ROS and apoptosis is not the enzyme’s active site. These data suggest that a vasculoprotective protein region could be derived from PRCP and utilized to promote vascular health, the kind of which would reduce thrombosis risk and its consequences. Thus, by having well-characterized mechanistic studies on animal models, we are able to tease out new proteins that have potential to target development for new kinds of agents to protect an organism from thrombosis without anticoagulation.

Thus, there are several challenges we should ask of investigators who develop new antithrombotics. First is to find new targets for agent development through careful mechanistic investigations in *in vivo* studies. It is clear that thrombosis risk is a polygenic problem and focus only in the classic hemostasis, anticoagulant, and fibrinolysis fields will develop agents that have variable risk for hemostatic defects. However, these *in vivo* systems do not exist in isolation and modifying genes and proteins may prove better antithrombotics with less or no bleeding risks. Additional challenges to investigators are finding resources to develop novel ideas in a world where there are decreasing funding for basic science with a translational goal. Finally, a challenge needs to be sent to pharmaceutical companies whose decision makers are inherently conservative and choose the obvious for drug development programs like all their competitors. It is very exciting that one oral agent has been approved for therapy venous thromboembolism in the United States and three other agents have been submitted for regulatory approval within the last 6 months. Yet all these agents have the same thrombin and factor Xa targets and all have a risk for interfering with hemostasis while protecting from thrombosis. Like governmental funding agencies, pharmaceutical companies need to expand their research and development into novel targets for thrombosis protection without bleeding risk. Developing these programs is the real challenge for the coming decade.

## Conflict of Interest Statement

The author declares that the research was conducted in the absence of any commercial or financial relationships that could be construed as a potential conflict of interest.
